# Targeted delivery of antisense oligonucleotides to hepatocytes using triantennary *N*-acetyl galactosamine improves potency 10-fold in mice

**DOI:** 10.1093/nar/gku531

**Published:** 2014-07-03

**Authors:** Thazha P. Prakash, Mark J. Graham, Jinghua Yu, Rick Carty, Audrey Low, Alfred Chappell, Karsten Schmidt, Chenguang Zhao, Mariam Aghajan, Heather F. Murray, Stan Riney, Sheri L. Booten, Susan F. Murray, Hans Gaus, Jeff Crosby, Walt F. Lima, Shuling Guo, Brett P. Monia, Eric E. Swayze, Punit P. Seth

**Affiliations:** Isis Pharmaceuticals, Inc., 2855 Gazelle Court, Carlsbad, CA 92010, USA

## Abstract

Triantennary *N*-acetyl galactosamine (GalNAc, **GN3**), a high-affinity ligand for the hepatocyte-specific asialoglycoprotein receptor (ASGPR), enhances the potency of second-generation gapmer antisense oligonucleotides (ASOs) 6–10-fold in mouse liver. When combined with next-generation ASO designs comprised of short *S*-cEt (*S*-2′-*O*-Et-2′,4′-bridged nucleic acid) gapmer ASOs, ∼60-fold enhancement in potency relative to the parent MOE (2′-*O*-methoxyethyl RNA) ASO was observed. **GN3**-conjugated ASOs showed high affinity for mouse ASGPR, which results in enhanced ASO delivery to hepatocytes versus non-parenchymal cells. After internalization into cells, the **GN3**-ASO conjugate is metabolized to liberate the parent ASO in the liver. No metabolism of the **GN3**-ASO conjugate was detected in plasma suggesting that **GN3** acts as a hepatocyte targeting prodrug that is detached from the ASO by metabolism after internalization into the liver. GalNAc conjugation also enhanced potency and duration of the effect of two ASOs targeting human apolipoprotein C-III and human transthyretin (TTR) in transgenic mice. The unconjugated ASOs are currently in late stage clinical trials for the treatment of familial chylomicronemia and TTR-mediated polyneuropathy. The ability to translate these observations in humans offers the potential to improve therapeutic index, reduce cost of therapy and support a monthly dosing schedule for therapeutic suppression of gene expression in the liver using ASOs.

## INTRODUCTION

Antisense oligonucleotides (ASOs) are short chemically modified oligonucleotides that bind to complementary RNA in cells via Watson-Crick base-pairing and modulate RNA function to produce a pharmacological effect (1). Second-generation gapmer ASOs are fully phosphorothioate (PS)-modified (2) chimeric ASOs with a central gap region of 8–14 PS DNA nucleotides flanked on either end with 2–5 2'-*O*-methoxyethyl RNA (MOE, Figure 1A) nucleotides (3). Second-generation ASOs (4) promote degradation of complementary RNA via the RNase H mechanism and represent the most advanced oligonucleotide therapeutic platform in the clinic. Kynamro, the first systemically delivered second-generation ASO targeting apolipoprotein B100 mRNA, was recently approved by the Food and Drug Administration (FDA) for the treatment of homozygous familial hypercholesterolemia (5). In addition, there are currently over 30 second-generation ASOs at various stages of clinical development for the treatment of cardiovascular, diabetes, cancer and several rare and orphan disease indications. Roughly half of these ASOs target genes that are predominantly expressed by hepatocytes in the liver—an organ that plays a central role in metabolism and in the synthesis and secretion of carrier proteins, coagulation factors, hormones and apolipoproteins (6). The liver is also a major site of accumulation of PS-modified ASOs (7). However, PS ASOs accumulate preferentially in the non-parenchymal (np) cells of the liver (8), which do not express majority of the gene targets of therapeutic interest.

*To whom correspondence should be addressed. Tel: +1 760 603 2587; Email: pseth@isisph.com

| ASO | Dose (mg/kg) | SRB1 mRNA $ED_{50}$ (mg/kg) |
|---|---|---|
| SRB1-M | 3, 10, 30 | 18.3 |
| GN3-SRB1-M | 0.5, 1.5, 5, 15 | 3.3 |
| SRB1-C | 0.7, 2, 7, 20 | 2.2 |
| GN3-SRB1-C | 0.07, 0.2, 0.7, 2, 7 | 0.3 |

**Figure 1.** (**A**) Structures of ASO chemical modifications and (**B**) triantennary *N*-acetyl galactosamine cluster (GN3). GalNAc conjugation enhances potency of 5-10-5 MOE (scavenger receptor B1-M, SRB1-M) and 2-10-2 cEt (SRB1-C) ASOs targeting SRB1 mRNA in mouse liver. (**C**) Dose–response curves for reducing mSRB1 mRNA in liver. Mice (C57BL/6, $n = 4$/group) were injected subcutaneously with a single dose of ASO (M = MOE, C = cEt) at the indicated doses and sacrificed after 72 h. Liver tissue was homogenized and analyzed for reduction in mSRB1 mRNA and normalized to saline-treated animals. (**D**) $ED_{50}$ (mg/kg) for reducing SRB1 mRNA in mouse liver. (**E**) GN3-SRB1-M ($K_i$ 14.6 nM) inhibits the binding of $^{125}$I-de-AGP to ASGPR in a competition binding assay. All data are expressed as mean ± SD.

Thus, strategies for targeted delivery of ASOs to hepatocytes are highly desirable and could enhance ASO potency for human therapy.

The asialoglycoprotein receptor (ASGPR), also known as the Ashwell receptor (9), is a C-type lectin that is abundantly expressed on hepatocytes (∼500 000 copies/cell) (10,11). The receptor is conserved across all mammals and clears serum glycoproteins by receptor-mediated endocytosis (12). The functional receptor is a trimer comprised of two proteins (HL-1 and HL-2) and exhibits high affinity for *N*-acetyl galactosamine (GalNAc) terminated oligosaccharides (13). Binding to the receptor is mediated by calcium (14) and is sensitive to pH, which facilitates dissociation of the ligand-receptor complex in acidic pH within endosomes and subsequent recycling of the receptor back to the cell surface (15). The structure activity relationships for ASGPR binding have been well established and a number of high-affinity triantennary synthetic ligands have been described (16–19).

The ASGPR has been utilized for the targeted delivery of modified lipoprotein particles (20), genes (21), chemically modified oligonucleotides (22,23), siRNA (24) and microRNA antagonists (25) to hepatocytes in rodents. Furthermore, GalNAc-conjugated siRNA targeting transthyretin (TTR) mRNAs, have shown good activity for reducing TTR protein in blood in early stage human trials (26,27). Given this background, we examined if GalNAc conjugation could be utilized for targeted delivery of second-generation ASOs to hepatocytes and if this could enhance ASO potency in animal models.

## MATERIALS AND METHODS

### Synthesis of triantennary *N*-acetyl galactosamine-conjugated ASOs

The GN3 (Figure 1B) loaded polystyrene solid support was prepared according to the general procedures described previously (19,24) and in Supplementary schemes S1–S6. ASOs (Table 1) were synthesized at 40-µmol scale using

UnyLinker$^{TM}$ support functionalized by modified nucleoside or GalNAc cluster. For the synthesis, 0.1 M solutions of all phosphoramidites in acetonitrile, and standard oxidizing and capping reagents were used. For each of the modified analogs, 4-fold excess of amidite was delivered with a 12-min coupling time. The 5′-end dimethoxytrityl group was left on to facilitate purification. Postsynthetically, all oligonucleotides were treated with 1:1 triethylamine:acetonitrile to remove cyanoethyl protecting groups from the PS linkages. Subsequently, oligonucleotides were treated with conc. aq. $NH_4OH$ at 55°C for 9–12 h to cleave from support, remove protecting groups and hydrolyze the UnyLinker$^{TM}$ moiety. Oligonucleotides were purified by ion-exchange chromatography using a gradient of NaBr across a column packed with Source 30Q resin. Pure fractions were desalted using high performance liquid chromatography (HPLC) on a reverse phase column. Purity and mass of oligonucleotides were determined using ion-pair Liquid chromatography mass spectrometry (LCMS) analysis (Table 1).

## Animal treatment

Animal experiments were conducted in accordance with the American Association for the Accreditation of Laboratory Animal Care guidelines and were approved by the Animal Welfare Committee (Cold Spring Harbor Laboratory's Institutional Animal Care and Use Committee guidelines). The animals were housed in microisolator cages on a constant 12-h light-dark cycle with controlled temperature and humidity and were given access to food and water *ad libitum*. Blood was collected by cardiac puncture exsanguination with $K_2$-ethylenediaminetetraacetic acid (EDTA) (Becton Dickinson Franklin Lakes, NJ, USA) and plasma separated by centrifugation at 10 000 rcf for 4 min at 4°C. Plasma transaminases were measured using a Beckman Coulter AU480 analyzer. Tissues were collected, weighed, flash frozen on liquid nitrogen and stored at −60°C. Reduction of target mRNA expression was determined by real-time polymerase chain reaction (RT-PCR) using 7700 RT-PCR sequence detector (Applied Biosystems). Briefly, RNA was extracted from about 50–100-mg tissue from each mouse using PureLink Pro 96 Total RNA Purification Kit (Life Technologies, Carlsbad, CA, USA) and mRNA was measured by qRT-PCR using Express One-Step SuperMix qRT-PCR Kit (Life Technologies). Primers and probes for the PCR reactions were obtained from Integrated DNA technologies. The assay is based on a target-specific probe labeled with a fluorescent reporter and quencher dyes at opposite ends. The probe is hydrolyzed through the 5′-exonuclease activity of Taq DNA polymerase, leading to an increasing fluorescence emission of the reporter dye that can be detected during the reaction. RNA transcripts were normalized to total RNA levels using RiboGreen, RNA Quantitation Reagent (Molecular Probes). RiboGreen is an ultrasensitive fluorescent nucleic acid stain, which when bound to RNA has a maximum excitation/emission at ∼500 nm/525 nm. Data are mean values +/- standard deviations.

## Scavenger receptor B1 mouse protocol

Six to eight-week-old C57BL/6 mice (Charles River Laboratories) were treated according to the indicated treatment schedules. The sequences of primers and probe used for mouse scavenger receptor B1 (SRB1) are 5'-TGACAACGACACCGTGTCCT-3' for the forward primer, 5'-ATGCGACTTGTCAGGCTGG-3' for the reverse primer and 5'-CGTGGAGAACCGCAGCCTCCATT-3' for the probe.

## FXI mouse protocol

Eight-week-old male Balb-c mice (Charles River Laboratories) were treated according to the indicated treatment schedules. The sequences of primers and probe used for mouse FXI were as follows: 5'-ACATGACAGGCGCGATCTCT-3' (forward), 5'-TCTAGGTTCACGTACACATCTTTGC-3' (reverse) and 5'-TTCCTTCAAGCAATGCCCTCAGCAATX-3' (probe). *FXI plasma protein levels*: Mouse FXI plasma protein levels were measured by sandwich enzyme immunoassay. Briefly, assay plates were coated with anti-FXI antibody (R&D systems), blocked with 2% bovine serum albumin (BSA) and incubated with diluted mouse Platelet-poor plasma (PPP). After extensive washes, FXI protein was detected by incubation with biotinylated anti-FXI antibody (R&D systems), followed by incubation with streptavidin-peroxidase conjugate and 3,3′,5,5′-Tetramethylbenzidine (TMB) substrate (Sigma). FXI plasma protein levels were calculated using serial dilutions of recombinant mouse FXI protein (R&D systems) or serial dilutions of normal mouse plasma.

## α-1 Antitrypsin mouse protocol

C57BL/6 mice (Charles River Laboratories) were treated according to the indicated treatment schedules. The sequences of primers and probe used for mouse α-1 antitrypsin (A1AT) were forward: 5'-TTCTGGCAGGCCTGTGTTG-3', reverse primer: 5'-ATCCTTCTGGGAGGTGTCTGTCT-3' and fluorescence probe: 5'-CCCCAGCTTTCTGGCTGAGGATGTTC-3'. Plasma A1AT levels protein was measured with an enzyme-linked immunosorbent assay method according to manufacturer's recommendations (Alpco 41-A1AMS-E01).

## ASGPR competition binding assay

Desialylation and $^{125}$I-Labeling of α1-acid glycoprotein (AGP) for Competition Assay in Mouse Primary Hepatocytes – 4 mg AGP (100 nmol AGP) was incubated in 50-mM sodium acetate buffer (pH5) with 1 U neuraminidase-agarose for 16 h at 37°C. Desialylation was confirmed >90% by either sialic acid assay or size exclusion chromatography (SEC). Iodination using iodine monochloride was achieved using a previously described procedure (28). One milliliter of desialylated α1-acid glycoprotein (de-AGP, 1 mg/ml) and 0.2 ml 1-M glycine in 0.25 M NaOH (pH 10) were added to a mixture of 10-mM iodine chloride solution (7 μl/mg protein), Na$^{125}$I (2.5 μl/mg protein) and 1

**Table 1.** Summary of ASO sequence and analytical data

| ASO | Motif | Sequence (5' to 3') | Mass Calcd. | Mass Obs. | % UV purity |
|---|---|---|---|---|---|
| SRB1-M | 5-10-5 | GCTTCAGTCATGACTTCCTT | 7164.2 | 7163.6 | 92.7 |
| GN3-SRB1-M | 5-10-5 | GCTTCAGTCATGACTTCCTTAGalNAc3 | 9238.4 | 9236.5 | 93.9 |
| SRB1-C | 2-10-2 | *TC*AGTCATGACT*TC* | 4646.95 | 4646.4 | 94.6 |
| GN3-SRB1-C | 2-10-2 | *TC*AGTCATGACT*TC*AGalNAc3 | 6721.08 | 6719.4 | 91.9 |
| FXI-M | 5-10-5 | TGGTAATCCACTTTCAGAGG | 7234.25 | 7232.69 | 98.4 |
| GN3-FXI-M | 5-10-5 | TGGTAATCCACTTTCAGAGGAGalNAc3 | 9211.98 | 9210.18 | 93.6 |
| A1AT-M | 5-10-5 | ACCCAATTCAGAAGGAAGGA | 7270.30 | 7269.52 | 95.6 |
| GN3-A1AT-M | 5-10-5 | ACCCAATTCAGAAGGAAGGAAGalNAc3 | 9344.44 | 9342.64 | 87.8 |
| ApoC3-M | 5-10-5 | AGCTTCTTGTCCAGCTTTAT | 7165.2 | 7164.3 | 95.1 |
| GN3-ApoC3-M | 5-10-5 | AGCTTCTTGTCCAGCTTTATAGalNAc3 | 9239.4 | 9238.7 | 96.2 |
| TTR-M | 5-10-5 | TCTTGGTTACATGAAATCCC | 7183.2 | 7182.2 | 90.5 |
| GN3-TTR-M | 5-10-5 | TCTTGGTTACATGAAATCCCAGalNAc3 | 9257.4 | 9255.4 | 92.7 |

Base code A = adenine, T = thymine, G = guanine, C = 5-methylcytosine. Upper case letters indicate DNA nucleotides; underlined letters indicate MOE nucleotides; underlined and italicized letters indicate S-cEt nucleotides. The 5-10-5 and 2-10-2 ASOs are fully PS modified, while the linker to GalNAc3 are phosphodiesters. A1AT, α-1 antitrypsin; SRB1, scavenger receptor B1.

M glycine in 0.25 M NaOH (25 μl/mg protein). After incubation for 10 min at room temperature, $^{125}$I -labeled de-AGP was separated from free $^{125}$I by concentrating the mixture twice utilizing a 3 kDa MWCO spin column. The protein was tested for labeling efficiency and purity on a HPLC system equipped with an Agilent SEC-3 column (7.8 × 300 mm) and a β-RAM counter.

Competition experiments utilizing $^{125}$I-labeled de-AGP and GN3-SRB1-M were performed as follows—freshly isolated mouse hepatocytes ($10^6$ cells/ml) were plated on six-well plates in 2 ml of appropriate growth media. The primary hepatocytes were cultured in William's media containing 10% fetal bovine serum (FBS), 1× non-essential amino acids and 1× sodium pyruvate. Cells were incubated 16–20 h at 37°C with 5 and 10% $CO_2$, respectively. Cells were washed with media without FBS prior to the experiment. Cells were incubated for 30 min at 37°C with 1-ml competition mix containing appropriate growth media with 2% FBS, $10^{-8}$ M $^{125}$I -labeled de-AGP and GalNAc-cluster containing ASOs at concentrations ranging from $10^{-11}$ to $10^{-5}$ M. Non-specific binding was determined in the presence of $10^{-2}$ M GalNAc sugar. Cells were washed twice with media without FBS to remove unbound $^{125}$I -labeled de-AGP and competitor GalNAc ASO. Cells were lysed using Qiagen's RLT buffer containing 1% β-mercaptoethanol. Lysates were transferred to round bottom assay tubes after a brief 10-min freeze/thaw cycle and assayed on a γ-counter. Non-specific binding was subtracted before dividing $^{125}$I protein counts by the value of the lowest GalNAc-ASO concentration counts. The inhibition curves were fitted according to a single site competition binding equation using a non-linear regression algorithm.

## Protocol for cell fractionation experiments

Dosing solutions were prepared in phosphate-buffered saline, sterile filtered and quantified. Mice (Balb-c) were given a single subcutaneous injection at the indicated doses. Mice were sacrificed 48 h after the injection. *Liver perfusion:* Mouse liver was perfused as previously described (8,29). Briefly, mice were anesthetized with an intraperitoneal injection of 0.1 ml per 10 g ketamine/xylazine. Inferior vena cava was catheterized and clamped. Liver was perfused with Hank's Balanced Salt Solution (Life Technologies) and mesenteric vessel was cut for drainage. Liver was subsequently perfused with collagenase (Roche). Following the perfusion, liver was removed and gently messaged through sterile nylon mesh. Cells were washed in Williams E (Life Technologies) containing 10% fetal calf serum, (4-(2-hydroxyethyl)-1-piperazineethanesulfonic acid) (HEPES), L-glutamine and antibiotic/antimycotic. *Cell separations:* Liver perfusions were performed as described above. A portion of the whole liver cell suspension was collected for the whole liver fraction. The fraction was spun at 450×g, washed with PBS containing 0.5% BSA and 2 mM EDTA (wash buffer), and pelleted. The hepatocyte and np fractions were separated as described previously with the following modifications (30). Whole liver cell suspension was spun at 50×g. The resulting hepatocyte pellet was washed, spun and run over a 30% percoll (GE Healthcare) gradient. A final wash was performed to remove residual percoll and cells were subsequently pelleted. The np cell suspension from original 50×g hepatocyte spin was spun twice more at 50×g to remove contaminating hepatocytes. The supernatant was then spun at 450×g to pellet all np cells. It was washed once before being placed over a 17.6% (4 ml) and 8.2% (4 ml) OptiPrep™ (Sigma) step gradient. The interface of the gradient (np cells) was washed in wash buffer and pelleted at 450×g. Cell concentration and viability were determined for all final purified cell fractions prior to cell pelleting. Oligonucleotide in the fractionated cells was quantified using protocols described previously (8,31).

## *APOC3* transgenic mouse protocol

Human *APOC3* transgenic mice were maintained on a normal chow diet 1 week prior to ApoC3-M intraperitoneal administration at various doses. At 48 h after the second dose, mice were sacrificed for human hepatic apoC-III mRNA, human plasma apoC-III and triglyceride quantification. *ApoC-III* primers were 5'-GCCGTGGCTGCCTGAG-3' and 5'-AGGAGCTCGCAGGATGGAT-3' and fluorescent probe: 5'-CCTCAATACCCCAAGTCCACCTGCC-3'. *ApoC-III plasma protein analysis and triglyceride measure:* Approximately, 100 μl of 1:5 diluted plasma was analyzed using an Olympus Clinical Analyzer and a commercially available turbidometric apoC-III assay (Kamiya, Cat# KAI-006). The assay protocol was performed as described by the vender. Plasma triglyceride levels were also quantified on the Olympus Clinical Analyzer using a Beckman Coulter Triglyceride assay (0SR61118).

### TTR transgenic mice protocol

TTR-Ile84Ser transgenic mice were generated and described previously (32). ASOs or vehicle PBS were injected subcutaneously with indicated dose for indicated periods. Animals were sacrificed 72 h after last dosing. Liver mRNA levels were analyzed as described above and plasma TTR levels and chemistry values were measured on the AU480 Clinical Chemistry Analyzer (Beckman Coulter, CA, USA). Human TTR primers were as follows: forward 5'-CCCTGCTGAGCCCCTACTC-3'; reverse: 5'-TCCCTCATTCCTTGGGATTG-3'; and fluorescence probe: 5'-ATTCCACCACGGCTGTCGTCA-3'.

### $ED_{50}$ determination

$ED_{50}$ values were determined with GraphPad Prism 5 software plotting log dose versus mRNA relative to untreated controls using a four-parameter fit with variable slope and constraining bottom = 0 and top = 1.

## RESULTS

### Combining next-generation chemical designs with GN3 conjugation improves potency ∼60-fold

We first examined if conjugating GN3 (24) (Figure 1B) to the 3'-terminus of ASO SRB1-M could enhance potency in mice in a single dose escalation experiment. SRB1-M is a fully PS-modified ASO with a 10-base DNA gap flanked on each end with 5 MOE nucleotides (abbrev. 5-10-5 MOE gapmer, Figure 1A and Table 1) targeting SRB1 mRNA. SRB1 is ubiquitously expressed in all cell types in the liver and in extrahepatic tissues and ASOs targeting mouse SRB1 have been previously used to understand the effect of chemical modifications and length on ASO activity in liver and in extrahepatic tissues in mice (31). In addition, we also tested the GN3 conjugate of SRB1-C, a next-generation 14-mer, which has the same 10-base DNA gap as SRB1-M and where two MOE nucleotides on each end of the gap have been replaced with *S*-2'-*O*-ethyl-2',4'-constrained bridged nucleic acids (33) (abbrev. 2-10-2 cEt gapmer, Figure 1A and Table 1). SRB1-C was previously shown to exhibit greater potency in mice than SRB1-M despite a lower $T_m$ and liver accumulation (31).

Mice (C57BL/6, $n$ = 4/group) were injected subcutaneously with a single dose of ASOs SRB1-M (3, 10 and 30 mg/kg), GN3-SRB1-M (0.5, 1.5, 5 and 15 mg/kg), SRB1-C (0.7, 2, 7 and 20 mg/kg) and GN3-SRB1-C (0.07, 0.2, 0.7, 2 and 7 mg/kg). Mice were sacrificed after 72 h; livers were homogenized and analyzed for reduction of SRB-1 mRNA. Both GN3 versions of ASOs SRB1-M and SRB1-C showed 6–7-fold enhanced potency relative to their parent ASOs and GN3-SRB1-C showed a 60-fold enhancement in potency relative to SRB1-M for reducing SRB-1 mRNA in mouse liver (Figure 1C and D). The improved potency was specific for liver as enhanced potency was not observed in tissues such as kidney and lung (data not shown). All ASOs were well tolerated with no elevations in plasma transaminases or organ weights (data not shown).

### GN3 conjugation improves potency of MOE ASO targeting mouse factor XI

We determined if GalNAc conjugation could improve potency of a 5-10-5 MOE ASO (FXI-M) targeting mouse coagulation factor XI (FXI) using a 3-week dosing schedule, which helps understand the effect of drug accumulation and metabolism on ASO activity. FXI is a member of the intrinsic coagulation pathway and is expressed specifically in hepatocytes and secreted in blood. Inhibiting FXI activity shows benefits in animal models of thrombosis with a minimal risk of bleeding (34,35). Mice (Balb-c, $n$ = 4/group) were injected subcutaneously with 1.5, 5 and 15 mg/kg of FXI-M and 0.35, 1 and 3 mg/kg of GN3-FXI-M twice weekly for 3 weeks. Mice were sacrificed 72 h after the last injection and liver tissues were homogenized and analyzed for reduction of mouse FXI mRNA. In addition, we also measured reductions in mouse FXI protein levels in plasma. GN3-FXI-M showed a 7-fold enhancement in potency for reducing FXI mRNA levels in liver and a 9-fold enhancement for reducing FXI protein in plasma (Figure 2A and B). Both ASOs were well tolerated with no elevations in serum transaminases (Figure 2C) or organ or body weight changes (data not shown).

### GN3 conjugation improves potency of MOE ASO targeting mouse A1AT

We also examined if GalNAc conjugation improves potency of a 5-10-5 MOE ASO (A1AT-M) targeting mouse A1AT in a 3-week dosing schedule. However, unlike the FXI study where the weekly dose was fractionated into two injections, ASOs were dosed once weekly to ascertain if dosing regimen could impact fold-change in potency. ASOs targeting A1AT were recently evaluated for the treatment of α-1 antitrypsin deficiency, which results from accumulation of mutant A1AT protein in liver (36). Mice (C57BL/6, 4/group) were injected subcutaneously with A1AT-M (5, 15 and 45 mg/kg) and GN3-A1AT-M (0.6, 2, 6 and 20 mg/kg) once a week for 3 weeks. Mice were sacrificed 72 h after the last injection and liver and plasma were analyzed for reductions in A1AT mRNA and protein, respectively (Figure 3A). We observed a dose-dependent reduction of the mRNA and protein in both groups, but GN3-A1AT-M showed a 10-fold enhancement in potency relative to A1AT-M (Figure 3B). Both ASOs were well tolerated with no elevations in plasma transaminases or blood urea nitrogen (BUN) (Figure 3D) and organ or body weights (Figure 3E).

To determine if GalNAc conjugation affected the duration of effect, mice (C57BL/6, $n$ = 4/group) were injected with a single dose of A1AT-M (100 mg/kg) or GN3-A1AT-M (20 mg/kg) and reduction of A1AT protein in plasma was measured by weekly tail bleeds (Figure 3C). At day 10 after injection, GN3-A1AT-M produced 80% reduction of protein in plasma with 40% suppression remaining at day 28. In contrast, A1AT-M resulted in 60% reduction of protein while nearly returning to baseline by day 28.

A

B

| $ED_{50}$ (mg/kg/wk) | mRNA | Protein |
|---|---|---|
| FXI-M | 8.8 | 9.5 |
| GN3-FXI-M | 1.3 | 1.1 |

C

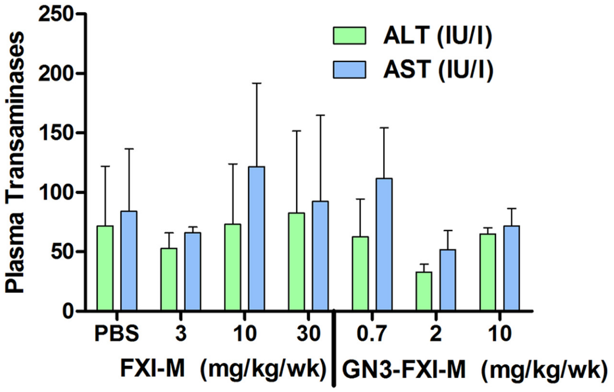

**Figure 2.** GalNAc conjugation enhances potency of mouse FXI ASO FXI-M. (**A**) Mice (Balb-c, $n = 4$/group) were injected subcutaneously with FXI-M (1.5, 5 and 15 mg/kg) and GN3-FXI-M (0.35, 1 and 5 mg/kg) twice weekly for 3 weeks. (**B**) $ED_{50}$ (mg/kg/wk) for reducing FXI mRNA in mouse liver and FXI protein in plasma. (**C**) FXI-M and GN3-FXI-M were well tolerated with no elevations in serum transaminases after 3 weeks of dosing. One animal from FXI-M (3 mg/kg group) was omitted because of anomalous transaminase reading. All data are expressed as mean ± SD. ALT, alanine aminotransferase; AST, aspartate aminotransferase.

## GN3 conjugation enhances ASGPR binding and increases total and fraction of administered ASO dose to hepatocytes

We determined the ASGPR-binding affinity of SRB1-M and GN3-SRB1-M by measuring their ability to compete with desialylated $^{125}$I-labeled α1-acid glycoprotein ($^{125}$I-deAGP), a high-affinity ASGPR ligand, for binding to isolated mouse hepatocytes (17) (Figure 1E). Briefly, AGP was desialylated using neuraminidase and labeled using Na$^{125}$I and iodine monochloride (28). Freshly isolated mouse primary hepatocytes were incubated with $^{125}$I-deAGP (10 nM) and SRB1-M or GN3-SRB1-M at concentrations ranging from $10^{-11}$ to $10^{-5}$ M. GN3-SRB1-M showed competitive inhibition of $^{125}$I-deAGP binding ($K_i$ = 14.6 nM), while SRB1-M showed no discernible competition ($K_i$ >100000 nM) clearly establishing the importance of GN3 for ASGPR binding and for improved potency.

To ascertain if the improved potency was a result of enhanced delivery of ASO to hepatocytes, mice (Balb-c, $n$ = 3/group) were injected subcutaneously with a single dose of SRB1-M (3, 10, 20 and 30 mg/kg) and GN3-SRB1-M (0.5, 1, 3, 6 and 18 mg/kg) and sacrificed after 48 h. Liver tissue from the treated animals was fractionated into hepatocytes and np cells and ASO accumulation in the different cell fractions was quantitated (8,29). We observed increased uptake of GN3-SRB1-M relative to SRB1-M in liver tissue (Figure 4A and B). Furthermore, >70% of SRB1-M in liver was found in the np cell fraction at all doses evaluated (Figure 4C). In comparison, >80% of GN3-SRB1-M in liver was found in the parenchymal cell fraction (Figure 4D), thus providing a rationale for the enhanced potency.

## GN3-ASO conjugate is metabolized to release parent ASO in the liver

The metabolic fate of the GN3 cluster after internalization into liver was determined in a time-course experiment. Mice (C57BL/6, $n = 4$/group) were injected subcutaneously with 10 mg/kg of GN3-SRB1-M and groups were sacrificed at 1, 2, 4, 8, 24 and 72 h after injection. Livers were homogenized and the ASO and metabolites were extracted and identified by LCMS (Supplementary Figure S1). Interestingly, very little intact GN3-SBR1-M was extracted from tissue even at the 1-h timepoint. The major metabolite was GN3-SRB1-M where the GalNAc sugars had been removed. Analysis of the plasma at this timepoint showed intact GN3-SRB1-M in plasma (data not shown), suggesting that metabolism occurs once the conjugate is internalized into the liver. The metabolites at later timepoints included those arising from hydrolysis of phosphodiester bonds between the ASO and GN3 and from hydrolysis of the amide linkages in GN3. No portion of GN3 was attached to the ASO at the 72-h timepoint, indicating that the ASO-GN3 conjugate is metabolized to liberate free ASO in the liver.

## Investigating GN3 conjugation to improve potency of ASOs in clinical development

We next examined if GalNAc conjugation could enhance the potency and duration of action of second-generation MOE ASOs targeting human apoC-III (ApoC3-M) and human TTR (TTR-M) in transgenic mouse models. ApoC3-M is currently being evaluated for the treatment of severe hypertriglyceridemia and familial chylomicronemia syndrome and has shown 70–80% reduction in apoC-III protein and serum triglycerides in multiple phase 2 clinical trials at a dose of 300 mg/week (∼4 mg/kg/week) (37). TTR-M is being evaluated for the treatment of TTR-associated polyneuropathy and has shown ∼75% reduction in TTR protein in a phase 1 trial at a dose of 300 mg/week (∼4 mg/kg/week) (38).

## GN3 conjugation enhances potency and duration of effect of ASO targeting human APOC-III in transgenic mice

Human *APOC3* transgenic mice ($n$ = 3/group) were injected subcutaneously with a single dose of ASOs ApoC3-M (1, 3, 10 and 30 mg/kg) and GN3-ApoC3-M (0.3, 1,

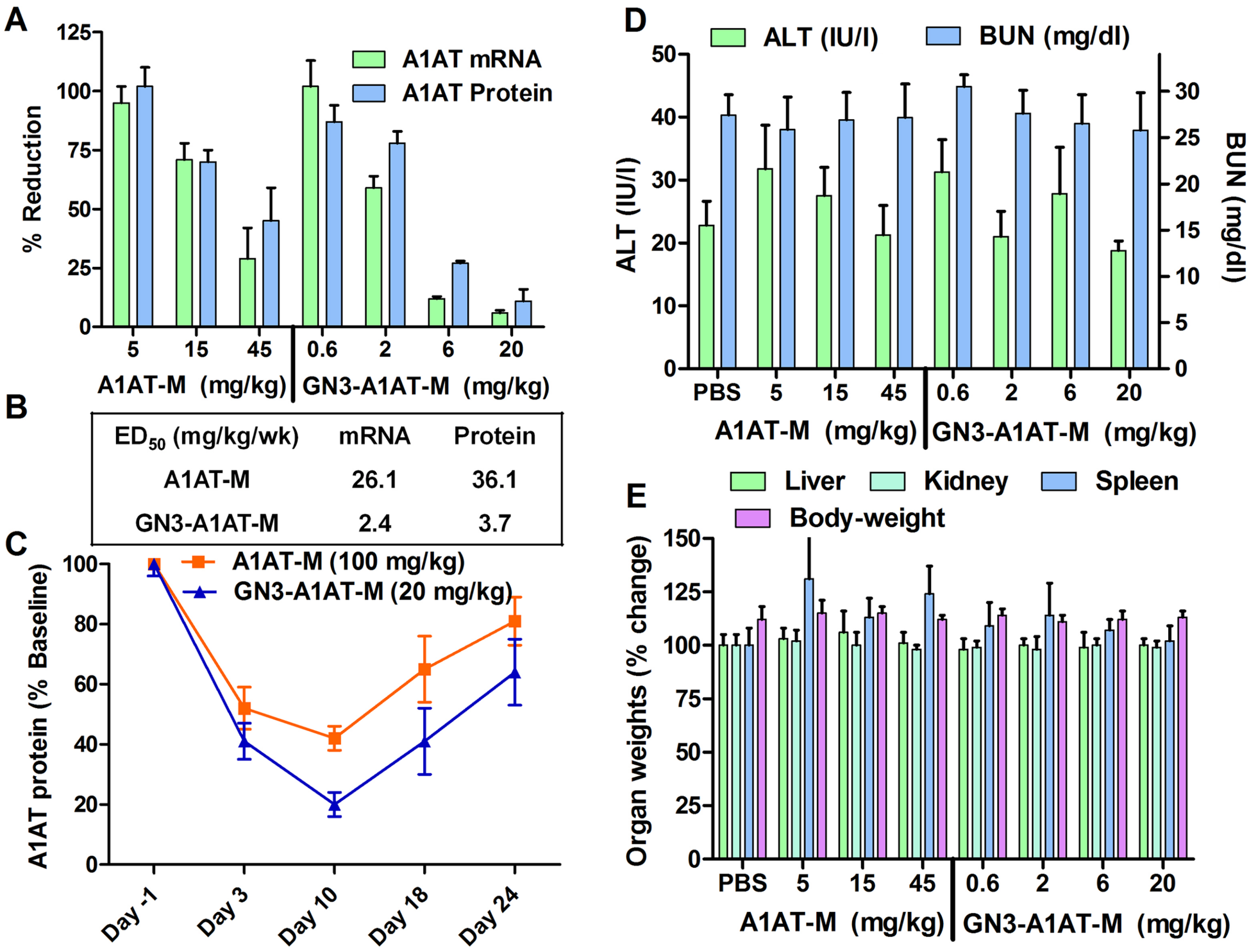

**Figure 3.** (A) Reduction of mouse A1AT mRNA in mouse (C57BL/6) liver and A1AT protein in plasma following subcutaneous injection of A1AT-M (5, 15 and 45 mg/kg) and GN3-TTR-M (0.6, 2, 6 and 20 mg/kg) once a week for 3 weeks. (**B**) $ED_{50}$ (mg/kg/wk) for reducing A1AT mRNA in mouse liver and protein in plasma. (**C**) Recovery of A1AT protein in mouse plasma following subcutaneous injection of A1AT-M (100 mg/kg) or GN3-A1AT-M (20 mg/kg). Tolerability data from the 3-week study showing no drug-induced elevations in (**D**) plasma transaminases or (**E**) organ weights. All data are expressed as mean ± SD.

3 and 10 mg/kg). Mice were sacrificed after 72 h and analyzed for reduction of human apoC-III mRNA in liver and for reductions in human apoC-III protein and total triglycerides in mouse plasma. GN3-ApoC3-M showed a 7-fold enhancement in potency for reducing human apoC-III mRNA in liver and an 11–13-fold enhancement for reducing human apoC-III protein and triglycerides in mouse plasma relative to ApoC3-M (Figure 5A and B). Both ASOs were well tolerated with no elevations in serum transaminase levels (Figure 5C). To ascertain if GalNAc conjugation affected duration of pharmacodynamic effect, transgenic mice were injected with a single dose of ApoC3-M (30 mg/kg) or GN3-ApoC3-M (10 mg/kg). Reductions in plasma apoC-III protein (Figure 5D) and triglycerides (Figure 5E) were monitored by weekly retro-orbital bleed over 6 weeks. For mice treated with ApoC3-M, recovery of plasma apoC-III protein and triglycerides plateaued by day 28. In contrast, mice treated with GN3-ApoC3-M displayed a slower recovery rate of apoC-III protein and plasma triglyceride levels, suggesting that GalNAc conjugation extends the ASO duration of effect.

#### GN3 conjugation enhances potency and duration of effect of ASO targeting human TTR in transgenic mice

Human transgenic mice (TTR-Ile84Ser, $n = 4$/group) were injected subcutaneously with TTR-M (6, 20 and 60 mg/kg) and GN3-TTR-M (0.6, 2, 6 and 20 mg/kg) once a week for 3 weeks. Mice were sacrificed 72 h after last injection and analyzed for reduction of hTTR mRNA in liver and for reduction of hTTR protein in mouse plasma relative to saline-treated control mice. GN3-TTR-M showed a 10-fold enhancement in potency for reducing hTTR mRNA in liver and hTTR protein in mouse plasma relative to TTR-M (Figure 6A and B). Both ASOs were well tolerated with no elevations in serum transaminase levels (Figure 6D) or organ weights (Figure 6E). To establish if the prolonged duration of effect observed in the apoCIII experiments was a general property, transgenic mice were injected with a single

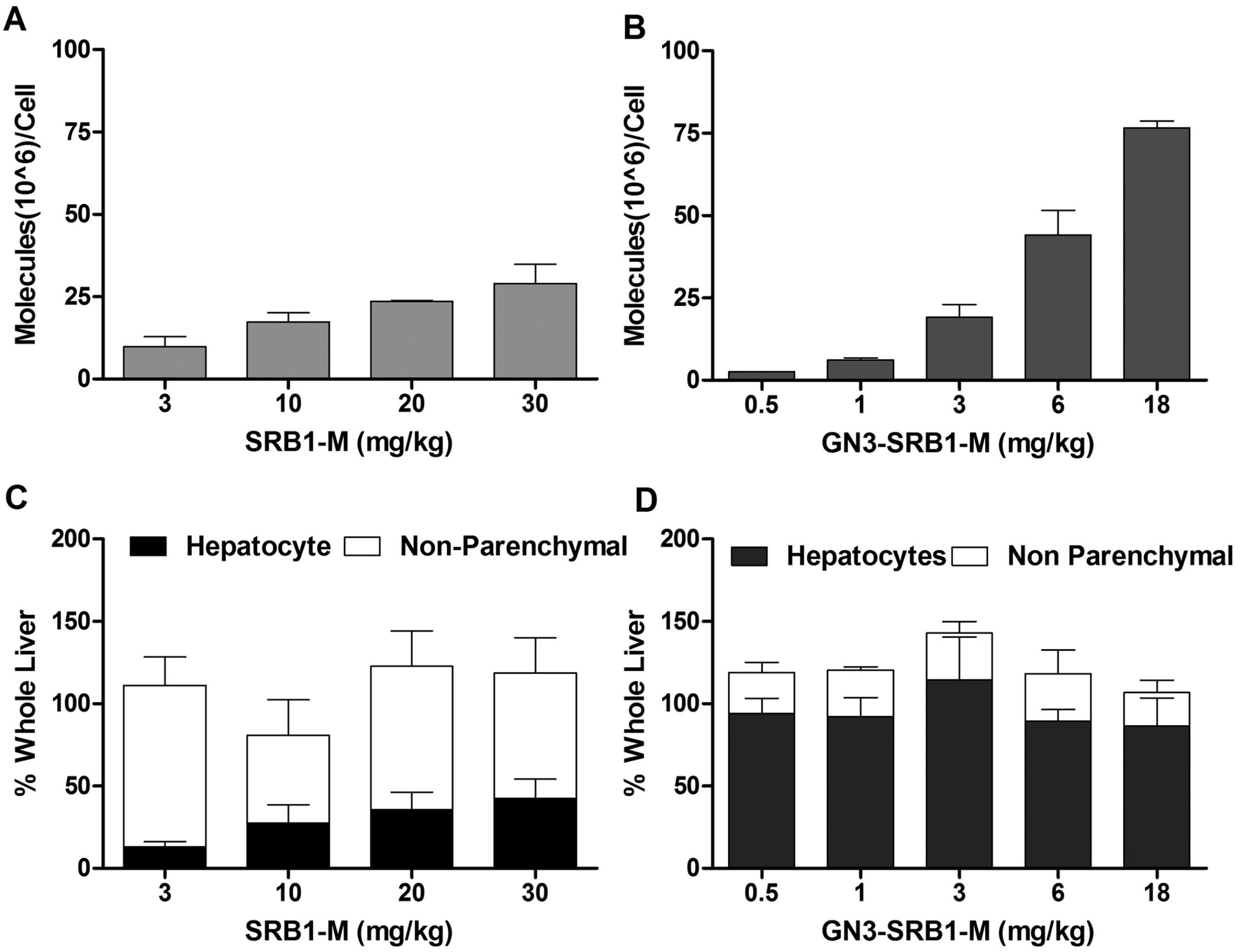

**Figure 4.** ASO distribution in mouse hepatocytes and np cells. Mice (Balb-c, $n = 3$/group) were injected subcutaneously with a single dose of SRB1-M (3, 10, 20 and 30 mg/kg) and GN3-SRB1-M (0.5, 1, 3, 6 and 18 mg/kg). Mice were sacrificed after 48 h; liver tissue was fractionated into hepatocytes and np cells and assayed for ASO accumulation. ASO molecules/cell in hepatocytes following injection of (**A**) SRB1-M or (**B**) GN3-SRB1-M ASOs. Differential accumulation of (**C**) SRB1-M and (**D**) GN3-SRB1-M between hepatocytes and np cells. All data are expressed as mean ± SD.

dose of TTR-M (100 mg/kg) or GN3-TTR-M (13.5 mg/kg) (Figure 6C). Reductions in plasma TTR protein were monitored by weekly tail bleeds for ∼7 weeks. For mice treated with TTR-M, plasma hTTR protein returned to baseline by day 39, while GN3-TTR-M-treated mice still retained ∼30% suppression of plasma hTTR protein levels 39 days post injection.

## DISCUSSION

Oligonucleotide-based therapeutics represent a third distinct drug discovery platform after small molecule and protein therapeutics. Gapmer ASOs exert their pharmacological effects via RNase H-mediated degradation of RNA and represent the leading platform for suppressing gene expression in human trials. Over 30 RNase H ASOs are in clinical development of which, approximately half target genes predominantly expressed by hepatocytes in the liver. We demonstrate that conjugation of a triantennary GalNAc cluster can improve potency of RNase H ASOs ∼10-fold for suppressing gene expression in the liver in wild-type and transgenic mouse models. GalNAc conjugation also results in an enhanced duration of pharmacological effect, which could support a monthly dosing schedule in human trials.

Unconjugated PS ASOs exhibit predictable pharmacokinetic/pharmacodynamic properties across multiple species, (39,40) and distribute broadly to all tissues (but do not cross the blood–brain barrier) after systemic administration with organs such as liver and kidney showing the highest levels of accumulation (7). After injection into an animal, PS ASOs bind to plasma proteins by virtue of their enhanced avidity for proteins. They circulate transiently in plasma before partitioning onto cell surface receptors. Internalization into cells is thought to occur via receptor-mediated endocytosis and multiple scavenger receptor pathways have been implicated (41,42).

The liver is comprised of parenchymal cells (hepatocytes), representing almost 80% of the total organ mass, and np

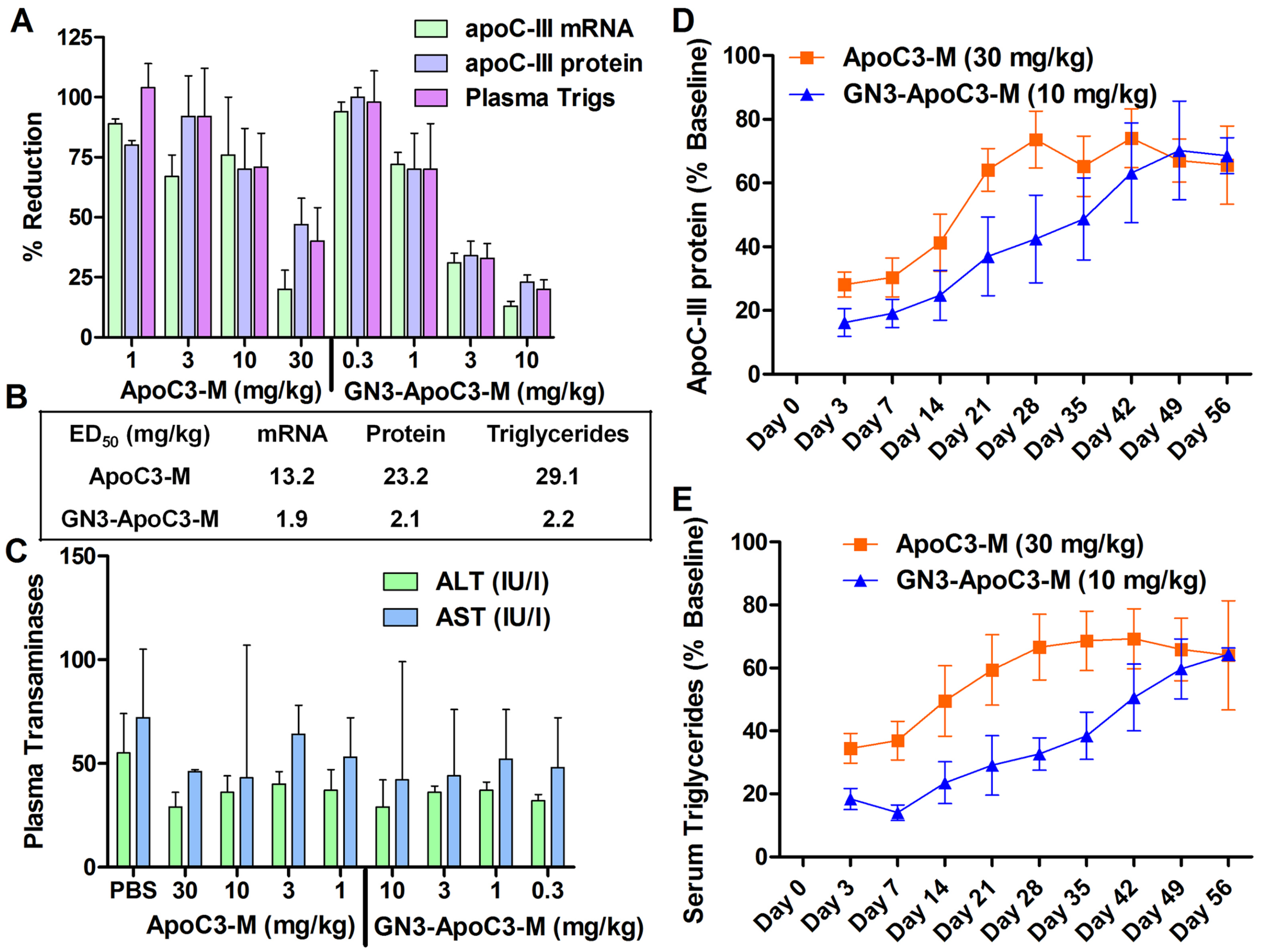

**Figure 5.** (**A**) Reduction of human apoC-III mRNA in transgenic mouse (C57BL/6, $n = 3$/group) liver and human apoC-III protein and triglycerides in plasma following intraperitoneal injection of a single dose of ApoC3-M (1, 3, 10 and 30 mg/kg) and GN3-ApoC3-M (0.3, 1, 3 and 10 mg/kg). (**B**) $ED_{50}$ (mg/kg) for reduction of human apoC-III mRNA in mouse liver and human apoC-III protein and triglycerides in plasma. (**C**) Plasma transaminase levels. Recovery of (**D**) human apoC-III protein and (**E**) triglycerides in mouse plasma following subcutaneous injection of ApoC3-M (30 mg/kg) or GN3-ApoC3-M (10 mg/kg). All data are expressed as average ± SEM, except for transaminases, which are expressed as mean ± SD.

cells that include sinusoidal enthothelial cells, Kupffer and stellate cells. Unconjugated PS ASOs accumulate preferentially in np cells of the liver with a smaller fraction being delivered to hepatocytes (41). Currently, a majority of genes of therapeutic interest are expressed in parenchymal cells of the liver. Thus, higher doses of PS ASOs are administered to saturate the high-capacity ASO-uptake sites in the np cells prior to drug accumulation in hepatocytes (8,29).

One strategy to deliver PS ASOs more effectively to hepatocytes is to target a high-affinity/high-capacity cell surface receptor, which is specific for hepatocytes (10,11). ASGPR is a C-type lectin, which is abundantly expressed on hepatocytes of all mammals (11). Its physiological role includes maintaining the homeostasis of serum glycoproteins (12) as well as, mitigating the lethal coagulopathy of sepsis (43). ASGPR exhibits high-affinity binding for GalNAc-terminated oligosaccharides (13). Upon ligand binding, the ASGPR-ligand complex is rapidly internalized into clathrin-coated pits and trafficked into early endosomes (10). In the acidic pH of endosomes, the receptor-ligand complex dissociates, the receptor is recycled back to the cell surface and the ligand is trafficked into lysosomes for degradation (15).

It is likely that similar ligand receptor binding and internalization dynamics are also involved in the uptake of GalNAc-conjugated ASOs into hepatocytes. However, the biological receptor for the ASOs (the targeted mRNA) resides in the nucleus or in the cytosol. Thus, the ASO has to escape the endosomal/lysosomal compartments to gain access to its biological receptor in order to produce a pharmacological effect. Given that ASOs are highly polar, negatively charged macromolecules, it is unlikely that they can exit these compartments by passive diffusion. Given these dynamics, a key unanswered question is how and when does the ASO gain access to the cytosol after being released into the endosomal compartments?

It is conceivable that membrane dynamics that occur during vesicle formation, produce discontinuities in the lipid bi-

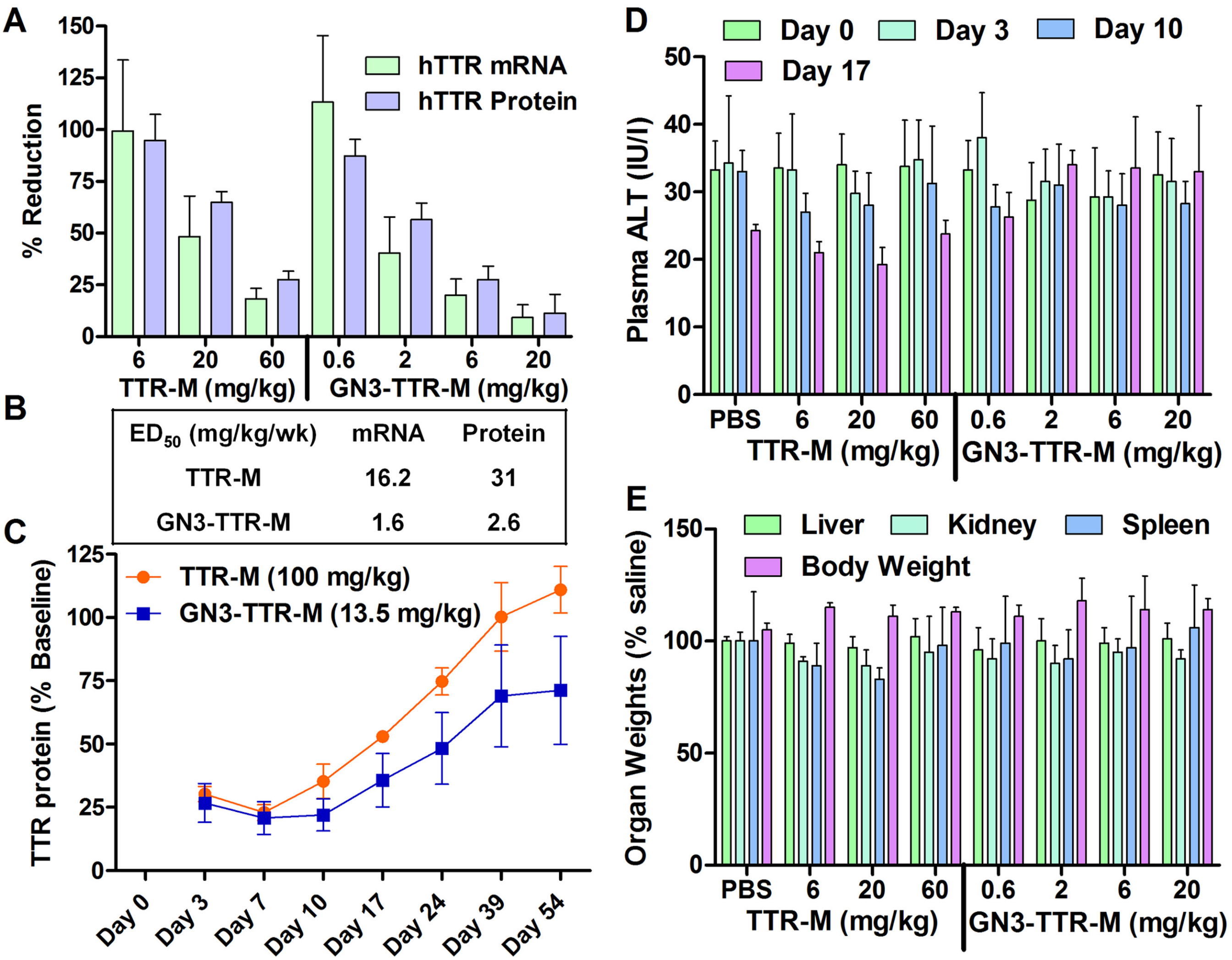

**Figure 6.** (**A**) Reduction of human TTR mRNA in transgenic mouse (TTR-Ile84Ser, $n = 4$/group) liver and hTTR protein in plasma following subcutaneous injection of TTR-M (1, 3, 10 and 30 mg/kg) and GN3-TTR-M (0.3, 1, 3 and 10 mg/kg) once a week for 3 weeks. (**B**) $ED_{50}$ (mg/kg/wk) for reduction of hTTR mRNA in mouse liver and protein in plasma. (**C**) Recovery of hTTR protein in mouse plasma following subcutaneous injection of TTR-M (100 mg/kg) or GN3-TTR-M (13.5 mg/kg). Tolerability data from the 3-week study showing no drug-induced elevations in (**D**) plasma transaminases or (**E**) organ weights. All data are expressed as mean ± SD.

layers providing an opportunity for some contents to escape into the cytosol (44). This hypothesis is strengthened by the exceptional activity seen with the shorter cEt modified ASO GN3-SRB1-C. Shorter ASOs modified with high-affinity nucleotides exhibit improved activity in mice, despite lower overall RNA-binding affinity and tissue accumulation (45–47). This is likely a result of lower molecular weight and reduced negative charge, which potentially facilitates ASO escape from endosomal compartments. Once in the cytosol, ASOs can gain access to nuclear compartments (48).

Previous studies have shown that conjugation of other tri-antennary GalNAc clusters to neutral methylphosphonate (22) or first-generation PS DNA (23) oligonucleotides can enhance accumulation in hepatocytes, but this did not lead to improved pharmacological inhibition. In this report, we used a previously identified ASGPR ligand (GN3), which has been adapted for attachment to oligonucleotides (24), to increase delivery of PS gapmer ASOs into hepatocytes. GN3-conjugated ASOs showed high affinity for mouse ASGPR, which results in enhanced ASO delivery to hepatocytes versus np cells. After internalization into cells, the GN3-ASO conjugate is metabolized at several sites to liberate the parent ASO in the liver. The sites of metabolism include glycosidic bonds of the GalNAc sugars, phosphodiester linkages between the ASO and the GN3 cluster, and amide linkages within the cluster. All the GN3 conjugate was metabolized to liberate the free ASO within 72 h. No metabolism of the ASO-GN3 conjugate was detected in plasma, suggesting that GN3 acts as a hepatocyte targeting prodrug, which is detached from the ASO by metabolism after internalization into the liver. However, while the GN3 cluster used in our studies is efficient for enhancing ASO potency, its synthesis is lengthy and somewhat cumbersome. Ongoing work in our laboratory will determine if simplifying the GN3 scaffold to facilitate ease of manufacturing will maintain or increase potency.

## CONCLUSION

We show that conjugation of a triantennary GalNAc cluster can improve the potency of second-generation gapmer oligonucleotides by approximately 10-fold in mouse models, when targeting genes expressed in hepatocytes. The enhanced potency appears translatable to all oligonucleotide sequences, as a direct result of enhanced delivery of the ASO to hepatocytes. For next-generation ASO designs, we observed a 60-fold enhancement in potency as compared to the parent second-generation 5-10-5 MOE ASO. Efforts are underway to fully characterize the PK/PD and toxicological properties of GalNAc-modified ASOs, in anticipation of progressing this platform toward human clinical trials. These enhancements in ASO potency and duration of action could significantly improve the therapeutic index, reduce therapy costs and support a monthly dosing schedule for suppression of hepatic gene expression in man.

## SUPPLEMENTARY DATA

Supplementary Data are available at NAR Online.

## FUNDING

Funding for open access charge: Isis Pharmaceuticals.
*Conflict of interest statement.* None declared.
